# CCC-GPU: A graphics processing unit (GPU)-optimized nonlinear correlation coefficient for large transcriptomic analyses

**DOI:** 10.1101/2025.06.03.657735

**Published:** 2025-06-06

**Authors:** Haoyu Zhang, Kevin Fotso, Milton Pividori

**Affiliations:** Department of Biomedical Informatics, University of Colorado Anschutz Medical Campus, Aurora, CO, USA; Office of Information Technology, University of Colorado Anschutz Medical Campus, Aurora, CO, USA; Department of Biomedical Informatics, University of Colorado School of Medicine, Aurora, CO 80045, USA; Colorado Center for Personalized Medicine, University of Colorado Anschutz Medical Campus, Aurora, CO, USA

## Abstract

**Motivation:**

Identifying meaningful patterns in complex biological data necessitates correlation coefficients capable of capturing diverse relationship types beyond simple linearity. Furthermore, efficient computational tools are crucial for handling the ever-increasing scale of biological datasets.

**Results:**

We introduce CCC-GPU, a high-performance, GPU-accelerated implementation of the Clustermatch Correlation Coefficient (CCC). CCC-GPU computes correlation coefficients for mixed data types, effectively detects non-linear relationships, and offers significant speed improvements over its predecessor.

**Availability and Implementation:**

CCC-GPU is openly available on GitHub (https://github.com/pivlab/ccc-gpu) and distributed under the BSD-2-Clause Plus Patent License.

## Introduction

Correlation coefficients are fundamental tools for uncovering meaningful patterns within data. While traditional measures like Pearson and Spearman are adept at capturing linear and monotonic relationships, newer methodologies, such as the Clustermatch Correlation Coefficient (CCC) [1] and the Maximal Information Coefficient (MIC) [2] have emerged to detect a broader spectrum of associations.

The CCC is a clustering-based statistic designed to identify not-only-linear patterns. We demonstrated CCC’s utility in analyzing gene expression data from GTEx, showcasing its robustness to outliers and its ability to detect both strong linear relationships and biologically significant non-linear patterns often missed by conventional coefficients. Unlike MIC, CCC offers the distinct advantage of accommodating both numerical and categorical data types and offers speeds up to a two orders of magnitude faster. However, despite leveraging CPU multi-threading for acceleration, the original CCC implementation can still be computationally intensive for large datasets. For instance, in the original study [1], we used only the top 5,000 most variables genes in a single tissue (whole blood) to reduce computation.

Here, we introduce a new implementation, CCC-GPU, that harnesses the power of NVIDIA CUDA for GPU programming. We have computed CCC-GPU values for all ~50,000 genes in GTEx across all 49 tissues in a fraction of the time that would have been needed using the original CCC implementation. This advancement achieves a substantial speedup over the original CPU-based implementation, making comprehensive correlation analysis of large biological datasets more practical and efficient.

## Methods

The initial version of CCC was developed entirely in Python. While numerous Python-based GPU acceleration libraries are now available, achieving the maximum flexibility and leveraging the complete feature set of CUDA often necessitates native C++ code [3]. Rather than undertaking a full re-implementation of CCC in C++, we opted for a strategic integration of the original Python codebase with high-performance C++ counterparts. This approach preserves the same Python interfaces and maintains the comprehensive feature set of the original implementation, ensuring a robust testing and evaluation cycle.

Through detailed profiling of the original CCC, we identified the internal computation of the Adjusted Rand Index (ARI) [1] as the primary performance bottleneck. To address this, we implemented the coefficient computation module in high-performance CUDA C++. This re-engineering enabled us to distribute the computationally intensive ARI calculations across the multiple parallel processing cores of one NVIDIA GPU. To seamlessly integrate this new CUDA C++ backend with the existing Python framework, we utilized pybind11 [4], a lightweight C++ library that exposes C++ functions and classes to Python. This ensures full compatibility and allows users to continue interacting with CCC-GPU via identical Python interfaces of CCC.

## Results

We conducted a comprehensive comparative evaluation of Pearson, Spearman, and CCC-GPU across all genes and tissues in the GTEx v8 dataset. The hardware used for the evaluation included an AMD Ryzen Threadripper 7960X CPU and an NVIDIA RTX 4090 GPU, representing a typical high-performance workstation configuration.

Our observations show that CCC-GPU achieves a considerable speedup compared to the original CCC, which was parallelized using 24 high-end CPU cores. Specifically, for the whole blood tissue dataset, comprising 56,200 genes and 755 samples, CCC-GPU exhibited a significant 42.5x speedup over the original CCC. This improvement effectively reduced the computation time for this specific analysis from 12 days to only 7 hours on a single machine. Similar substantial performance gains were consistently observed across other tissues, highlighting the broad applicability of CCC-GPU’s acceleration.

**Figure 1:**
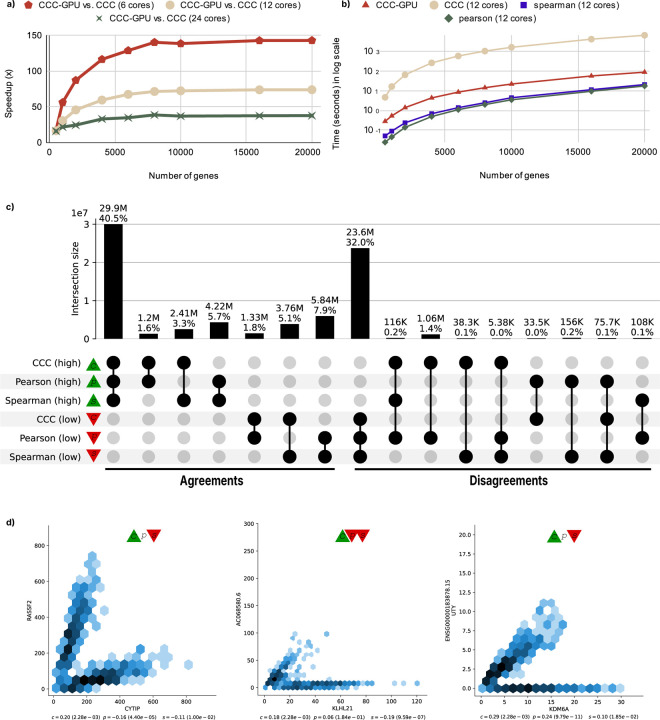
**a)** CCC-GPU’s performance comparison: speedup of CCC-GPU against the original CPU-based CCC, illustrating its scalability as the number of genes increases (with 1,000 samples fixed) and across different CPU core configurations. Further details are available in Supplementary Table S1. **b)** Comparative runtime of correlation methods: a direct comparison of execution times for Pearson, Spearman, the original CCC, and CCC-GPU. This panel shows how CCC-GPU significantly closes the performance gap with simpler methods, even with increasing gene numbers (fixed 1,000 samples and 12 CPU cores). Consult Supplementary Table S2 for more information. **c)** Agreements and disagreements among top/bottom correlations: UpSet plot visualizing the overlap and unique detections within the top 30% (high, green triangle) and bottom 30% (low, red triangle) correlation values identified by each coefficient. This highlights areas of consensus and unique insights. **d)** Non-linear pattern detection by CCC: hexagonal binning plots featuring illustrative gene pairs where CCC (c) reveals non-linear patterns that may be missed by Pearson (p) and/or Spearman (s). Green triangles indicate correlations in the top 30th percentile, red in the bottom 30th, while the absence of a triangle indicates values within the middle 30th-70th percentile range. Statistical significance is derived using the methodology from the original CCC paper.

To further understand CCC-GPU’s scalability with increasing data size, we performed benchmarks using synthesized input, comparing its speedup against the original CPU implementation. The results, illustrated in Figure 1a, demonstrate that CCC-GPU keeps a stable trend of speedup as input size increases, strongly confirming its robust scaling capabilities when handling large datasets. The different curves in Figure 1a simulate CCC-GPU’s prospective speedup over CPU hardware with varying numbers of cores.

We also collected and compared the runtime of the original CCC, CCC-GPU, Pearson, and Spearman methods, as shown in Figure 1b. While Pearson and Spearman are inherently faster due to them being based on simple statistics, CCC-GPU’s runtime is remarkably closer to these methods, showcasing its efficiency while maintaining its advanced capabilities for capturing complex relationships. This dramatic reduction in computation time transforms formerly intractable analyses into feasible and routine operations for researchers.

The significant performance enhancement provided by CCC-GPU expands the scope of transcriptomic analyses. With its enhanced speed, it is possible to uncover more non-linear patterns by applying our approach comprehensively to all genes across all tissues in large datasets efficiently. In the UpSet analysis [5] in Figure 1c, we compared Pearson, Spearman and CCC-GPU on how they agreed or disagreed in prioritizing gene pairs. We found that 1.2 million gene pairs (Disagreements in Figure 1c, where CCC-GPU is “high” and any of the others “low”) likely have nonlinear patterns. In Figure 1d, we show a subset of these gene pairs where the nonlinear pattern has a biological explanation, such as UTY-KDM6A (explained by sex differences) and RASSF2-CYTIP (explained by pre- and post-mortem status, and replicated in independent datasets [1]).

## Conclusions

We present CCC-GPU, a GPU-accelerated implementation of the original Clustermatch Correlation Coefficient (CCC). Our work demonstrates that CCC-GPU delivers a remarkable acceleration over its CPU-based predecessor, enabling the rapid and efficient computation of correlation coefficients in large transcriptomic datasets. This performance leap transforms analyses that previously required weeks into tasks achievable within hours on standard research hardware.

Challenges often reside not only in detecting but also in interpreting complex, non-linear links between genes. CCC’s ability to also correlate different data types allows researchers to easily incorporate metadata into their analyses. For example, a previously highlighted non-linear relationship for the gene pair RASSF2-CYTIP, detected by CCC, was explained by the metadata variable “COHORT” (pre- vs. post-mortem status). However, applying CCC to large datasets presented a significant computational bottleneck. CCC-GPU directly addresses that while preserving CCC’s full functionality, making the method much more efficient and broadly feasible.

Our new CCC-GPU delivers a next-generation correlation coefficient at a fraction of the computational cost without sacrificing its accessibility, accuracy or reliability. This significant performance enhancement makes comprehensive correlation analysis of large genomic data practical on standard research hardware. Beyond standard correlation analyses, CCC-GPU empowers biologists to perform sophisticated tasks such as advanced feature selection prior to machine learning model training with large datasets. Moreover, by accelerating the discovery of novel non-linear relationships in expression data, CCC-GPU helps researchers quickly identify patterns beyond conventional, linear-only relationships, potentially uncovering new biological insights that would otherwise remain hidden and driving forward our understanding of complex biological systems.

## Supplementary Material

1
