# Supplementary material for: CCC-GPU: A graphics processing unit (GPU)-optimized nonlinear correlation coefficient for large transcriptomic analyses
Source: bioRxiv. 2025 Jun 6:2025.06.03.657735. Preprint. [Version 1] doi: 10.1101/2025.06.03.657735 (PMC12157546; doi:10.1101/2025.06.03.657735)
Supplement: Supplementary file 1 [file NIHPP2025.06.03.657735v1-supplement-1.pdf]

bioRxiv preprint doi: https://doi.org/10.1101/2025.06.03.657735; this version posted June 6, 2025. The copyright holder for this preprint (which was not certified by peer review) is the author/funder, who has granted bioRxiv a license to display the preprint in perpetuity. It is made available under aCC-BY 4.0 International license.

Supplementary Information

Tables

Table S1: Speedup using different CPU configurations (1,000 fixed samples).

Number of genes	CCC-GPU vs. CCC (6 cores)	CCC-GPU vs. CCC (12 cores)	CCC-GPU vs. CCC (24 cores)
500	17.6x	16.52x	16.1x
1,000	56.17x	30.65x	21.82x
2,000	87.06x	45.72x	24.45x
4,000	116.39x	59.46x	33.03x
6,000	128.74x	67.46x	34.77x
8,000	140.24x	71.48x	38.67x
10,000	138.51x	72.38x	37.02x
16,000	142.7x	73.83x	37.53x
20,000	142.83x	73.88x	37.73x

Table S2: Execution time of different methods (1,000 fixed samples).

Number of genes	CCC-GPU	CCC (12 cores)	Spearman (12 cores)	Pearson (12 cores)
500	0.279s	4.603s	0.051s	0.024s
1,000	0.529s	16.198s	0.089s	0.045s
2,000	1.384s	63.263s	0.235s	0.138s
4,000	4.290s	255.071s	0.679s	0.489s
6,000	8.441s	569.409s	1.379s	1.093s
8,000	14.116s	1009.069s	2.443s	1.973s
10,000	21.694s	1570.269s	4.379s	3.448s
16,000	55.469s	4051.114s	11.169s	9.310s
20,000	86.286s	6374.781s	20.282s	17.238s
